# Disseminated nontuberculous mycobacterial infection with multifocal retinitis and vasculitis in an immunocompromised patient with anti-IFN-ɣ autoantibodies

**DOI:** 10.1186/s12348-016-0106-z

**Published:** 2016-10-22

**Authors:** Tian Loon Lee, Rupesh Agrawal, Jackie Yu-Ling Tan, Kiat Hoe Ong, Chen Seong Wong, Su Ling Ho

**Affiliations:** 1 National Healthcare Group Eye Institute, Tan Tock Seng Hospital, Singapore, 308433 Singapore; 2 National Healthcare Group, Department of General Medicine, Tan Tock Seng Hospital, Singapore, Singapore; 3 National Healthcare Group, Department of Haematology, Tan Tock Seng Hospital, Singapore, Singapore; 4 National Healthcare Group, Department of Infectious Disease, Tan Tock Seng Hospital, Singapore, Singapore

## Abstract

**Background:**

Nontuberculous mycobacteria (NTM) are found ubiquitously in the environment. Since exposure to NTM is universal, infection likely represents underlying host susceptibility factors. Anti-IFN-ɣ autoantibodies have been described previously in patients with NTM. Up to 88 % of patients with disseminated NTM or other opportunistic infections have high-titer anti-IFN-ɣ autoantibodies, compared with 2 % of patients with TB and healthy controls.

**Findings:**

We report a unique presentation of a patient with anti-IFN-ɣ autoantibodies with disseminated NTM infection who presents with panuveitis with multifocal retinitis and vasculitis. Treatment with systemic anti tubercular therapy resulted in complete clinical resolution with good visual recovery.

**Conclusions:**

Patients with anti-IFN-ɣ autoantibodies present with a novel syndrome that links autoimmunity and immunodeficiency. This case emphasizes the importance of testing for anti-IFN-ɣ autoantibodies in patients with disseminated mycobacterial infection.

## Background

Nontuberculous mycobacteria (NTM) are defined as mycobacteria other than *Mycobacterium tuberculosis* (MTB). NTM are found ubiquitously in the environment in soil, dust, and water [1, 2]. NTM were once thought to be nonpathogenic but are now known to cause a wide variety of human diseases including infections of the eye [3]. Since exposure to NTM is universal, infection likely represents underlying host susceptibility factors [4]. Genetic defects have been identified, predominantly in the setting of disseminated NTM infection, with numerous defects involving the type 1 cytokine pathway [5–8].

Despite its ubiquitous nature, NTM infections are still relatively uncommon. A review of 174 published case reports and series on NTM ocular infections found that the most frequently reported type of infection caused by NTM was keratitis (290 of 420 eyes; 69 %). On the other hand, the reported incidence of NTM infections causing choroiditis were rare (6 of 420 eyes; 1.5 %), as was the incidence of panuveitis from NTM infections (2 of 420 eyes; 0.5 %) [9].

Anti-IFN-ɣ autoantibodies have been described previously in patients with tuberculosis (TB) and human immunodeficiency virus (HIV) [10, 11], as well as disseminated mycobacterial and other infections [12, 13].

We report a unique case of a patient with anti-IFN-ɣ autoantibodies with disseminated NTM infection who presents with panuveitis with multifocal retinitis and vasculitis.

## Findings

### Case presentation

A 55-year-old Malay female presented with bilateral blurring of vision for 2 weeks associated with fever. Her best-corrected Snellen’s visual acuity was 6/21 in the right eye and 6/7.5 in the left eye. Pupils were equal and reactive to light with no relative afferent pupillary defect. The intraocular pressure was 17 mmHg in both eyes. On slit lamp examination, the conjunctiva was mildly injected, with anterior chamber (AC) cells of grade 2+ and flare of grade 1+ in both eyes, with a Koeppe nodule noted on the left iris. Both lens had nuclear sclerotic cataract.

**Fig. 1:**
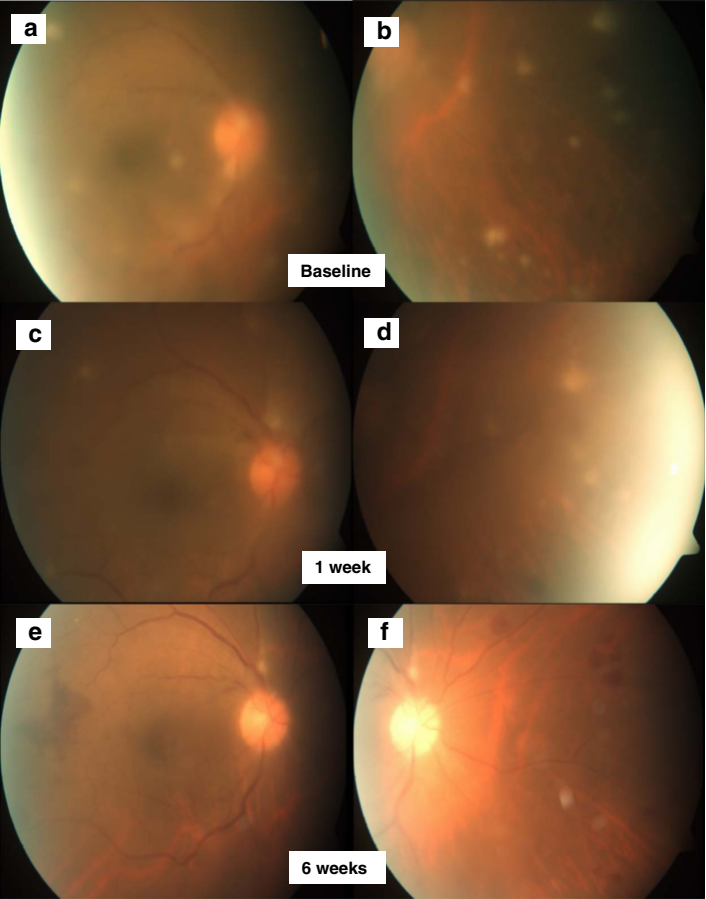
Right fundus photographs. Colour fundus photography demonstrates the presence of peripheral vascular sheathing with vitreous and retinal infiltrates at baseline in the posterior pole (**a**) and nasal retina (**b**). Decreasing vitritis and activity of the retinal infiltrates at week 1 in the posterior pole (**c**) and nasal retina (**d**) as well as further improvement at week 6 in the posterior pole (**e**) and nasal retina (**f**)

**Fig. 2:**
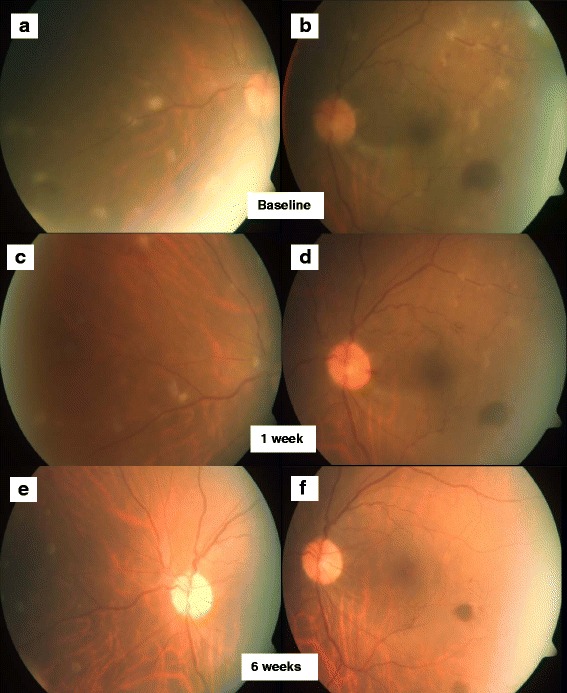
Left fundus photographs. Colour fundus photography demonstrates the presence of peripheral vascular sheathing with vitreous and retinal infiltrates at baseline in the posterior pole (**a**) and nasal retina (**b**). Decreasing vitritis and activity of the retinal infiltrates at week 1 in the posterior pole (**c**) and nasal retina (**d**) as well as further improvement at week 6 posterior pole (**e**) and nasal retina (**f**)

Posterior segment examination revealed vitritis, which was more marked in the right eye. The optic discs in both eyes were swollen. The macula appeared unremarkable in both eyes with no evidence of cystoid macular edema. There was peripheral vascular sheathing with vitreous and retinal infiltrates in both eyes (Figs. 1a,b and 2a,b).

A review of her past medical history revealed that two and a half years ago, she had been diagnosed with pulmonary TB and *Mycobacterium foruitum* (*M. fortuitum*) lymphadenitis when she presented with prolonged fever, cough, weight loss, and generalized lymphadenopathy. Sputum cultures were positive for MTB and she completed 9 months of treatment with rifampicin, isoniazid, and ethambutol. A biopsy of her right axillary lymph node grew *M. fortuitum* sensitive to moxifloxacin, amikacin, ciprofloxacin, and cotrimoxazole. She received induction therapy with 6 weeks of intravenous amikacin followed by 9 months of oral trimethoprim/sulphamethoxazole and levofloxacin.

A second biopsy of the right cervical lymph node was performed and reported suppurative granulomatous lymphadenitis with a few acid fast bacilli. However, the polymerase chain reaction test (PCR) was negative for TB bacilli.

After taking oral trimethoprim/sulphamethoxazole and levofloxacin for 9 months, she defaulted her medications for 3 months and represented with pyrexia and weight loss. The third and latest biopsy of the right inguinal lymph node grew *Mycobacterium abscessus* (*M. abscessus*) sensitive to amikacin and clarithromycin. This was accompanied by positive blood cultures for *M. abscessus*. She received a further 6 weeks of induction therapy with intravenous amikacin and cefoxitin, followed by oral levofloxacin, trimethoprim/sulphamethoxazole, and clarithromycin. No MTB treatment was given.

After 1 year of oral treatment for *M. abscessus*, she decided to stop her medication after a discussion with her infectious diseases physician citing pill burden and financial concerns. Three months later, she represented with her current complaints of pyrexia and bilateral blurring of vision.

**Fig. 3:**
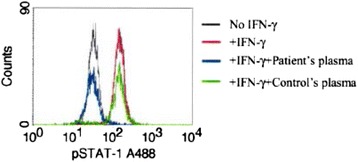
Report. IFN-ɣ stimulation of STAT-1 phosphorylation. Peripheral blood mononuclear cells were stimulated with IFN-ɣ and the level of phospho-STAT1 measured by flow cytometry in culture media (*red histogram*), the presence of patient’s plasma (*blue histogram*), or control plasma (*green histogram*)

She was seronegative for HIV and had normal CD4:CD8 counts. Her syphilis rapid plasma reagin and syphilis immunoglobulin G tests were negative. Further investigations found that she tested positive for anti-IFN-ɣ autoantibodies, which completely inhibited downstream phosphorylation of STAT-1 by IFN-ɣ (Fig. 3).

**Fig. 4:**
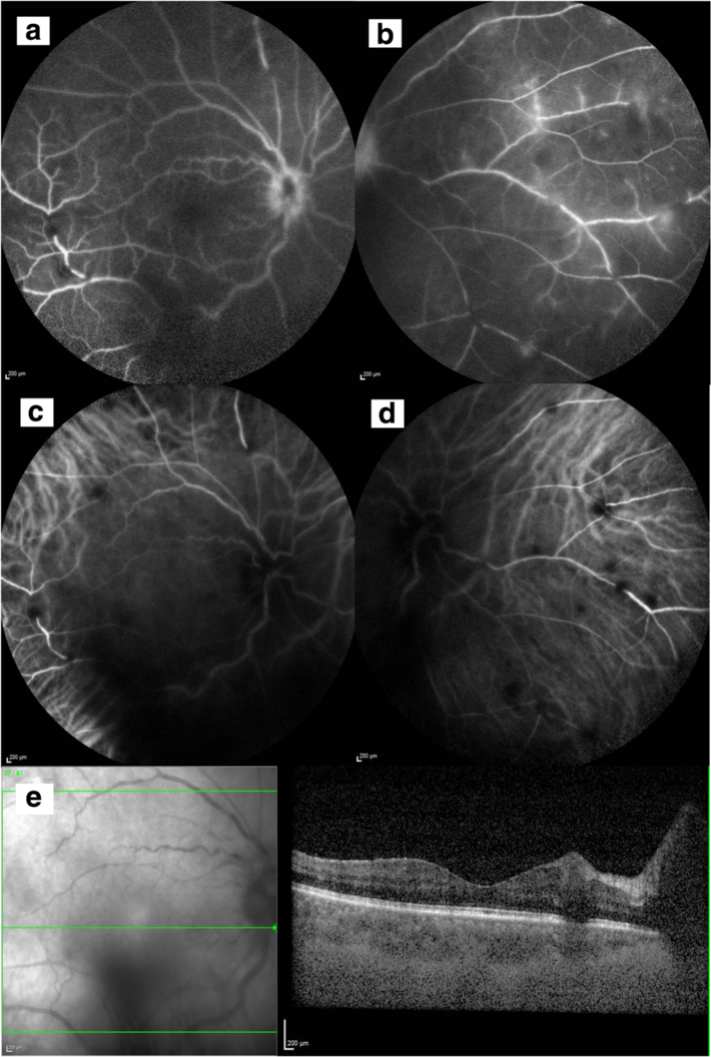
Baseline investigations of the right eye. Fundus fluorescein angiogram disc leakage (**a**) and peripheral perivascular leakage (**b**). Indocyanine green angiography showing patches of hypocyanescence corresponding to the blocked fluorescence from retinal infiltrates (**c**) and nasal retina (**d**). Spectral domain optical coherence tomography (SD OCT) shows a normal foveal contour with no cystoid macular edema with focal areas of retinal thickening corresponding to the retinal infiltrates on clinical examination (**e**)

**Fig. 5:**
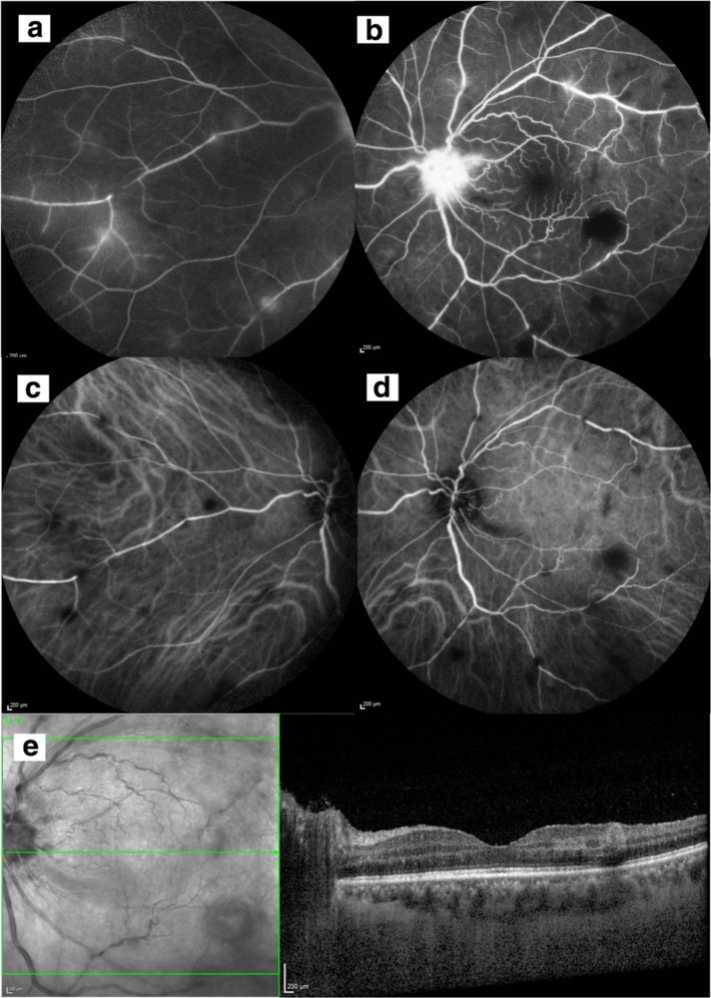
Baseline investigations of the left eye. Fundus fluorescein angiogram disc leakage (**a**) and peripheral perivascular leakage (**b**). Indocyanine green angiography showing patches of hypocyanescence corresponding to the blocked fluorescence from retinal infiltrates (**c**) and nasal retina (**d**). Spectral domain optical coherence tomography (SD OCT) shows a normal foveal contour with no cystoid macular edema with focal areas of retinal thickening corresponding to the retinal infiltrates on clinical examination (**e**)

Widefield fundus fluorescein angiogram (FFA) showed bilateral disc leakage and peripheral perivascular leakage (Figs. 4a,b and 5a,b). Indocyanine green angiography (ICG) showed patches of hypocyanescence corresponding to the blocked fluorescence from retinal infiltrates (Figs. 4c,d and 5c, d). Spectral domain optical coherence tomography (SD OCT) showed a normal foveal contour with no cystoid macular edema with focal areas of retinal thickening corresponding to the retinal infiltrates on clinical examination (Figs. 4e and 5e). A second computed tomography scan of her neck, thorax, abdomen, and pelvis showed that some of her abdominal and para-aortic lymph nodes had increased in size.

With the above clinical history and findings, and her history of recurrent presentations of pyrexia 3 months after stopping NTM treatment, the patient was diagnosed with panuveitis with multifocal retinitis and vasculitis likely secondary to NTM infection with a background of anti-IFN-ɣ autoantibodies. Besides topical steroids and antibiotic eye drops, the patient was started on empirical treatment for *M. abscessus.* This included 6 weeks of induction therapy with intravenous amikacin, intravenous cefoxitin, and oral clarithromycin. In view of her history of MTB and the presence of bilateral panuveitis, the decision was made to treat empirically for MTB until blood and acid fast bacilli cultures returned. She was started on oral rifampicin, isoniazid, and pyrazinamide 1 day after initiating NTM induction therapy. No form of oral or parenteral corticosteroid was given.

Over the course of a week, she reported improvement in her vision especially in her right eye to 6/12. Examination of both eyes revealed improvement in anterior chamber cells to a grade 1+, with decreasing vitritis and activity of the retinal infiltrates (Figs. 1c,d and 2c,d). Her fever also resolved after 2 days of treatment. She declined a vitreous tap opting to continue treatment for *M. abscessus* for 6 weeks and MTB for 8 weeks.

After 6 weeks, her vision had improved to 6/7.5 on the right eye and 6/6 on the left eye. No AC cells were noted. An examination of the posterior segment showed only mild disc swelling with no vitritis and the retinal infiltrates had resolved (Figs. 1e,f and 2e,f). She did not report any fever, weight loss, or malaise. Her NTM treatment was changed to oral trimethoprim/sulphamethoxazole, levofloxacin, and clarithromycin. A repeat computed tomography of the neck, thorax, abdomen, and pelvis reported that the previously prominent para-aortic lymph nodes were smaller in size.

After 8 weeks, all repeat blood and acid fast bacilli cultures returned negative results. In the absence of evidence of MTB, MTB treatment was stopped and she was continued on long term NTM treatment. Since then, the patient has had no recurrences of pyrexia, weight loss, or panuveitis while on NTM treatment. She was offered treatment with rituximab for her anti-IFN-ɣ autoantibodies, but declined.

### Discussion

Patel et al. reported finding anti-IFN-ɣ autoantibodies in 6 of 35 patients with disseminated mycobacterial infection and all 6 patients were females of East Asian descent [4]. These autoantibodies were high-titer, able to block binding of native human interferon-ɣ (IFN-ɣ), inhibitory to early aspects of IFN-ɣ signal transduction (STAT-1 phosphorylation), and inhibitory to some of the downstream biological consequences of IFN-ɣ binding (IFN-ɣ dependent upregulation of TNF-α and IL-12 production) [4]. Whether these autoantibodies are the cause or effect of the disseminated MAC infection is still unclear, although a study by Chi et al. on previously healthy Chinese adults, suggests that anti-IFN-ɣ autoantibodies may be the cause of the disseminated NTM infection [14].

More recently, Brown et al. reported high-titer anti-IFN-ɣ autoantibodies in 88 % of patients with disseminated NTM or other opportunistic infections compared to 2 % of patients with TB and healthy controls amongst patients from Thailand and Taiwan. This may indicate distinctive roles for IFN-ɣ in the control of different mycobacterial species [15]. The paucity of anti-IFN-ɣ autoantibodies in patients with TB alone suggests that mycobacterial infection itself does not lead to the development of anti-IFN-ɣ autoantibodies [15]. Similarly, patients with isolated pulmonary NTM do not have anti-IFN-ɣ autoantibodies suggesting that mycobacterial defence is also organ specific [15]. Both authors also found that in patients with anti-IFN-ɣ autoantibodies, the NTM were predominantly due to rapidly growing mycobacteria [4, 15]. Rapidly growing mycobacteria include *M. abscessus*, *M. fortuitum*, and *M. chelonae*. A recent study in 2013 which analysed NTM isolates from respiratory specimens of 20,182 patients from 62 laboratories across 6 continents found that amongst the rapid growers, *M. abscessus* and *M. fortuitum* were the most commonly isolated species worldwide. Rapid growing mycobacteria were found to be highly prevalent in East Asia when compared to the rest of the world [16].

In a review of 174 case reports and series on NTM ocular infections published in 2015, Kheir et al. found that of the 420 eyes concerned, 9 presented with uveitis (6 cases of choroiditis, 1 case of iridocyclitis, and 2 cases of panuveitis). Of these 9 patients, 5 had HIV/AIDS, 3 had disseminated NTM infection, and 1 was secondary to prior cataract and vitrectomy operation. No mention was made regarding the cause of the disseminated NTM in the 3 patients presenting with this infection [9]. As such, to our knowledge, this is the first reported case of a patient with anti-IFN-ɣ autoantibodies presenting with panuveitis with multifocal retinitis and vasculitis with a background of disseminated NTM infection.

We note that our patient was first diagnosed with MTB, prior to the isolation of other NTM. Her underlying anti-IFN-ɣ autoantibodies could have made her more susceptible to MTB. Indeed studies have shown a link between anti-IFN-ɣ autoantibodies and MTB infection [17, 18]. We concluded that given the history of treated MTB and the recent diagnoses of recurrent, disseminated NTM, our diagnosis of ocular NTM, without taking any vitreous samples for staining and cultures, was a reasonable one. Nevertheless, upon developing ocular involvement, the patient was treated systemically with targeted therapy to both NTM and MTB, without any systemic steroids. Clinical response was good, with the patient reporting improvement within a week and notable deterioration in the activity of the retinal lesions. After 6 weeks of treatment with anti-tuberculous therapy alone, there was a total resolution of ocular lesions.

The fact that our patient has had multiple admissions and treatment for NTM underlines the fact that such infections tend to be recurrent in these patient populations. Close ophthalmic follow-up is imperative to detect future recurrences of ocular involvement and treatment to prevent any sequelae.

There were small case series of successful treatment of this condition with the anti-CD20 monoclonal antibody, rituximab. Browne et al. [19] reported the treatment of four similar patients with multiple doses of rituximab, resulting in the decline of the pathogenic auto-antibodies, the recovery of IFN-ɣ-induced pSTAT-1 signalling, and most importantly, the reduction in the incidence of clinical infections. This patient was offered treatment with rituximab but for personal reasons had declined this option of treatment.

### Conclusion

Patients with anti-IFN-ɣ autoantibodies present with a novel syndrome that links autoimmunity and immunodeficiency. This case emphasizes the importance of testing for anti-IFN-ɣ autoantibodies in patients with disseminated mycobacterial infection. Systemic anti tubercular therapy resulted in complete clinical resolution with good visual recovery.
